# Micronutrient Deficiencies, Over- and Undernutrition, and Their Contribution to Anemia in Azerbaijani Preschool Children and Non-Pregnant Women of Reproductive Age

**DOI:** 10.3390/nu10101483

**Published:** 2018-10-11

**Authors:** James P. Wirth, Tamerlan Rajabov, Nicolai Petry, Bradley A. Woodruff, Nafisa Binte Shafique, Rashed Mustafa, Vilma Qahoush Tyler, Fabian Rohner

**Affiliations:** 1 GroundWork, 7306 Fläsch, Switzerland; nico@groundworkhealth.org (N.P.); woody@groundworkhealth.org (B.A.W.); fabian@groundworkhealth.org (F.R.); 2 UNICEF, 1095 Baku, Azerbaijan; trajabov@unicef.org (T.R.); nbshafique@unicef.org (N.B.S.); rmustafa@unicef.org (R.M.); 3 UNICEF Regional Office for Middle East and North Africa, Amman 11821, Jordan; vtyler@unicef.org

**Keywords:** Azerbaijan, anemia determinants, micronutrients, iron deficiency, undernutrition, overnutrition

## Abstract

Data on the nutritional situation and prevalence of micronutrient deficiencies in Azerbaijan are scarce, and knowledge about anemia risk factors is needed for national and regional policymakers. A nationally representative cross-sectional survey was conducted to assess the prevalence of micronutrient deficiencies, over- and undernutrition, and to disentangle determinants of anemia in children and women in Azerbaijan. The survey generated estimates of micronutrient deficiency and growth indicators for children aged 0–59 months of age (6–59 months for blood biomarkers) and non-pregnant women 15–49 years of age. Questionnaire data, anthropometric measurements, and blood samples were collected to assess the prevalence of under- and over-nutrition, anemia, iron deficiency, and iron deficiency anemia, in both groups. In children only, vitamin A deficiency and zinc deficiency were also assessed. In women only, folate and vitamin B12 deficiencies and vitamin A insufficiency were assessed. In total, 3926 household interviews were successfully completed with a response rate of 80.6%. In the 1455 children, infant and young child feeding practices were relatively poor overall; the prevalence of wasting and stunting were 3.1% and 18.0%, respectively; and 14.1% of children were overweight or obese. The prevalence of anemia was 24.2% in 6–59 months old children, the prevalence of iron deficiency was 15.0% in this age group, and the prevalence of iron deficiency anemia was 6.5%. Vitamin A deficiency was found in 8.0% of children, and zinc deficiency was found in 10.7%. Data from 3089 non-pregnant women 15–49 years of age showed that while undernutrition was scarce, 53% were overweight or obese, with increasing prevalence with increasing age. Anemia affected 38.2% of the women, iron deficiency 34.1% and iron deficiency anemia 23.8%. Vitamin A insufficiency was found in 10.5% of women. Folate and vitamin B12 deficiency were somewhat more common, with prevalence rates of 35.0% and 19.7%, respectively. The main risk factors for anemia in children were recent lower respiratory infection, inflammation and iron deficiency. In women, the main risk factors for anemia were iron deficiency and vitamin A insufficiency. Anemia is a public health problem in Azerbaijani children and women, and additional efforts are needed to reduce anemia in both groups.

## 1. Introduction

Globally, anemia affects about 40% of children under 5 years of age and 30% of non-pregnant women [1]. Anemia can cause reduced work capacity, poor pregnancy outcomes, increased maternal and perinatal mortality and morbidity, impaired cognitive performance and poorer educational achievement [2]. Its causes are multifactorial, and a recent model of anemia determinants estimated that the risk factors of anemia in Eastern Europe and Central Asia included micronutrient deficiencies, inflammation, and hemoglobinopathies, with iron deficiency speculated as the major cause [3].

Iron, vitamin A and zinc deficiencies are amongst the most prevalent micronutrient deficiencies globally, affecting all population groups, but particularly young children and women of reproductive age [4]. Iron, vitamin A and zinc deficiencies often occur concomitantly and substantially contribute to the burden of disease, in particular in young children [5]. Milder forms of single or multiple micronutrient deficiencies can lead to increased morbidity and mortality as well as impaired cognitive and psychomotor development [4].

Although micronutrient deficiencies negatively affect health during the whole life cycle, an adequate supply of iron, zinc and vitamin A during the first 1000 days of life—the time between conception and the child’s 2nd birthday are the most crucial in terms of development, since some developmental and functional delays during that period are not reversible or are only partly reversible [5]. Deficiencies of folate and vitamin B12 can result in megaloblastic anemia [6,7], yet there is only scant population-based data examining the association between these deficiencies and anemia [7]. Folate status is particularly important for women of reproductive age due to folate’s pre-conceptional role in preventing neural tube defects [8,9].

Data on micronutrient deficiencies in Azerbaijan are scarce; Azerbaijan’s Nutrition Survey (AzNS) in 2013 [10] collected the first nationally-representative data on micronutrient deficiencies in preschool age children and non-pregnant women 15–49 years old. The AzNS also collected anthropometric data, which enabled the exploration of the association between anemia and under- and over-nutrition in children and women. Understanding the linkages between anemia determinants is key to understanding the pathways linking anemia and its various risk factors. Understanding the linkages between overweight and obesity and other risk factors for anemia is of particular interest in Azerbaijan, as about one out of seven children and one in two women are considered overweight or obese [10].

## 2. Materials and Methods

### 2.1. Survey Design and Participants

The AzNS 2013 was conducted between February and April 2013 across Azerbaijan’s nine accessible economic regions (i.e., Baku, Absheron, Ganja-Gazakh, Shaki-Zaqatala, Lankaran, Guba-Khachmaz, Aran, Yukhari Karabakh, and Dakhlik Shirvan); each economic region was considered a separate survey stratum. The regions of Nakhchevan and Kalbajar-Lachin were not included in the sampling frame due to security concerns. As no socioeconomic, health, or nutrition data is available for Nakhchevan and Kalbajar-Lachin, it is not possible to determine if their exclusion resulted in biased national prevalence estimates for key indicators. In addition to stratum-specific estimates, after appropriate statistical weighting, estimates were calculated for urban and rural residence and for Azerbaijan as a whole. Within each stratum, first stage sampling selected 30 census enumeration areas (EA) with probability proportional to population size. From a recently compiled list of all households in each selected enumeration area, 16 households were selected with simple random sampling. All preschool-age children from each selected household were recruited for the survey. Women 15–49 years of age were recruited in two out of three selected households.

### 2.2. Data Collection and Laboratory Analysis

Experienced field workers received intense training in all data collection procedures. The household interview was administered first to the head of the household, if present. If not, any knowledgeable adult household member could be interviewed. The household questionnaire contained modules related to household demographics; socio-demographic variables; and water, sanitation and hygiene.

Short questionnaires were administered to the caretaker of all recruited preschool children (0–59 months of age) and to all recruited non-pregnant women. The child questionnaire collected information on infant and young child feeding practices, recent illnesses, recent consumption of iron or vitamin A supplements, and healthcare-seeking behavior of the caregiver for the child in question. Regarding recent illnesses, the child’s caretaker reported if s/he had diarrhea, fever, or cough in the 2 weeks preceding the interview. Children with concurrent cough, fever, and “difficulty breathing due to problem in chest” were classified as having an acute lower respiratory infection (LRI). The women questionnaire sought information on marital, pregnancy, and occupational status; obstetric history; ante- and perinatal care for the most recently born child; use of and attitudes toward iodized salt and fortified flour; and recent iron, vitamin A or folic acid supplement consumption. This manuscript concerns only indicators of nutritional status and other variables potentially associated with these indicators.

Anthropometric measurements were done on all children 0–59 months of age, and blood samples were collected from children 6–59 months of age. Anthropometric measurements and blood samples were collected from all non-pregnant women enrolled in the AzNS. Weight was measured using bathroom scales (LAICA, Barbarano Vicentino VI, Italy). If the child could stand quietly on the scale, they were weighed directly on the scale. If not, weight was measured using the scale’s tare function by taking the difference between the caretaker’s weight and the combined weight of the child and caretaker. Children were weighed with only light clothing. In children, length/height measurements were taken using wooden height/length boards (UNICEF, Copenhagen, Denmark); for those younger than 2 years old, length was measured with the child lying down. Height measurements were taken from women using a portable stadiometer (213 Portable Stadiometer, Seca, Hamburg, Germany).

For children, approximately 4 mL of venous blood was drawn into trace-element-free K2 EDTA-treated evacuated tubes (Vacutainer, Beckton Dickinson, Franklin Lakes, NJ, USA). For women, approximately 5 mL of venous blood was drawn into K2 EDTA-treated evacuated tubes (Vacuette^®^, Greiner Bio-One GmbH, Kremsmünster, Austria). Hemoglobin concentration was measured on-site using a portable hemoglobinopmeter (Hb201+, HemoCue™, Angelsholm, Sweden). Quality control of the HemoCue™ devices was conducted and recorded on a daily basis using control materials.

Labeled blood tubes containing whole blood were placed in cool boxes equipped with temperature data loggers to ensure a temperature between +3 °C and +8 °C in the field and during daily transportation to the Research Institute of Haematology and Transfusiology of the Azerbaijan Ministry of Health. Samples were processed either on the same day or in the morning of the following day, so that samples from all but three clusters were processed within 24 h after collection; for three very remote clusters, the time from blood sampling to processing was less than 30 h. Samples were centrifuged at 3000 rpm for 7 min to separate the plasma from the erythrocytes, platelets, and leukocytes, and then aliquoted into appropriately labeled cryovials. Aliquots were stored at −20 °C until shipped on dry ice to international laboratories.

Plasma for children and women was analyzed in one run for retinol-binding protein (RBP), ferritin, C-reactive protein (CRP) and a1-acid glycoprotein (AGP) at the VitMin-Lab (Wilstaett, Germany) using an ELISA method [11]. Plasma zinc concentration was measured in children only using inductively coupled plasma-optical emission spectrometry at the Center for Nutrition and Metabolism at the Children’s Hospital Oakland Research Institute (Oakland, CA, USA). To prevent zinc contamination, powder-free gloves were used during the blood draws and when processing the samples. In addition, all aliquots were prepared under a ventilation hood. To check for potential contamination, the blood sampling and processing procedures were mimicked using Ultrapure^®^ water collected and stored using the phlebotomy supplies employed in the survey (i.e., butterfly needles, trace element-certified vacutainers, disposable pipettes, and cryotubes). No zinc contamination was observed. Plasma from non-pregnant women was sent to the Swiss Vitamin Institute (Lausanne, Switzerland) for analysis of plasma folate and vitamin B12 concentrations. Folate concentrations were measured using a microbiological assay method with *Lactobacillus caseii* (ATCC 7469) as the test organism [12]. Vitamin B12 concentrations were measured on about ½ of all samples that were selected randomly using the AOAC Official Method 952.20 with *Lactobacillus leichmanii* as test organism.

### 2.3. Parameters and Clinical Thresholds

Hemoglobin concentrations were adjusted for elevation and, for women, smoking status using World Health Organization (WHO) guidelines [13]. Following this adjustment, children were classified as anemic if they had hemoglobin concentrations less than 110 g/L, and concentrations <70, 70–99, and 100–109 g/L denoted severe, moderate, and mild anemia, respectively. Hemoglobin concentrations less than 120 g/L were used to classify non-pregnant women as anemic, with concentrations of <80, 80–109, and 110–119 g/L denoting severe, moderate and mild anemia, respectively [13]. Using the weighted anemia prevalence in children and women in all strata combined, WHO criteria [13] were used to assess the public health problem posed by anemia in children and women in Azerbaijan.

Ferritin concentrations were adjusted for inflammation using each subject’s CRP and AGP values applying the method developed by Thurnham [14]. Ferritin concentrations <12 μg/L and <15 μg/L were used to define iron deficiency in children and women, respectively [15]. Individuals with concurrent anemia and iron deficiency were classified as having “iron deficiency anemia”. CRP and AGP were also used to adjust each subject’s RBP levels by applying the internal correction factor developed by Thurnham [16] for retinol, as retinol and RBP have been shown to be highly correlated [17]. In children, vitamin A deficiency was defined as RBP <0.7 µM/L, and in women, vitamin A insufficiency was defined as RBP <1.05 µM/L [18,19].

Cut-offs for elevated CRP and AGP were >5 mg/L and >1 g/L, respectively. The level of inflammation was categorized into four groups: no inflammation, elevated CRP only, elevated CRP and AGP, and elevated AGP only [20]. For bivariate and multi-variate analyses, a dichotomous variable was calculated to illustrate if an individual had either elevated CRP and/or AGP.

In children, zinc deficiency was defined using the non-fasting cutoffs of 65 µg/dL and 57 µg/dL depending if phlebotomy took place in the morning or afternoon, respectively [21]. A plasma folate concentration of <10.0 nmol/L was used to define folate deficiency according to WHO guidelines [4]. Vitamin B12 deficiency in women was defined as <150 pmol/L [22].

Height-for-age and weight-for-height were calculated for all children using the WHO growth standard [23], Z-scores below −2.0 were used to classify a child as stunted or wasted. Overweight in children was defined as a weight-for-height z-score greater than +2.0 but less than or equal to +3.0. Obesity was defined as a weight-for-height z-score greater than +3.0.

Chronic energy deficiency and overnutrition in non-pregnant women was assessed using body mass index (BMI; kg/m^2^). Cut-off points for BMI were as follows: <16.0 severe chronic energy deficiency; 16.0–16.9 moderate chronic energy deficiency; 17.0–18.4 at-risk for energy deficiency; 18.5–24.9 normal; 25.0–29.9 overweight; ≥30.0 obese [24].

### 2.4. Data Management and Statistical Analysis

Data were double entered into CSPro v. 5.0 with data-entry screens programmed to accept only codes within a predetermined range. Data analysis was done using SPSS version 21.0 with the complex survey module. Statistical weights for household variables were calculated in several steps to account for real and potential sampling biases. Statistical weights were used to correct for the different selection probability of the different sized strata; the stratum-specific weight applied to the households in each selected EA was adjusted up or down by the proportion of households found in that EA; and adjustment of the inverse of the complement of the proportion of households with non-response due to short-term absence or refusal was done.

Household characteristics and the prevalence of nutritional and micronutrient deficiencies were calculated in aggregate (i.e., for the entire sample across all strata), and means and medians were calculated for continuous variables. The statistical precision of all prevalence estimates was assessed using 95% confidence limits (95% CI) which were calculated accounting for complex sampling, including the cluster and stratified sampling used in this survey. The anemia prevalence of children and women was calculated for demographic subgroups and nutritional characteristics to identify potential risk factors of anemia. The statistical significance of differences between subgroups was assessed using Chi-square adjusted for the unequal probability of selection and complex sampling, and *p* values <0.05 were considered statistically significant.

To identify risk factors of anemia, variables significantly associated with anemia in children and women were identified separately using bivariate analyses. Priority was given to variables collected in the majority of subjects. As such, associations between anemia and complementary feeding indicators calculated for children 6–23 months of age were examined using bivariate analyses and were not considered for multivariate analyses. Separate poisson multivariate regression models incorporating robust sandwich variance [25] were then calculated for children and women. All variables statistically significantly associated with anemia in bivariate analyses were incorporated into the initial model. Those not contributing with statistical significance to the model were sequentially removed until only statistically significant factors remained. For variables remaining in the model, crude unweighted risk ratios of anemia for each variable were calculated separately. These regression models produced adjusted risk ratios (aRR) for each variable, which were used with the proportion of anemia cases exposed to the risk factor (pd) to calculate the population attributable fraction (PAF) of each risk factor for anemia using the following equation as recommended by Rockhill and colleagues [26]:

(1) $$\mathrm{pd}\frac{\left(\mathrm{aRR}-1\right)}{\left(\mathrm{aRR}\right)}$$

### 2.5. Ethics and Consent

All survey materials were reviewed and approved by Azerbaijan’s Ministry of Health and the Office of the President. Written informed consent was sought from the head of each household (or spouse in case of absence) on behalf of the children and eligible women within that household. For children and women, separate informed oral consent was sought from each eligible woman in the household and the mother or guardian of each eligible child.

Survey respondents diagnosed with severe anemia were referred for further diagnosis and treatment at the local health facility. No blood was taken from children younger than 6 months of age to avoid injury and undue stress.

## 3. Results

The AzNS successfully gathered data from 3926 (80.6%) of 4320 households selected for participation [10]. As shown in Table 1, about one-half of these 3926 household were located in urban areas, and three-quarters of households were headed by males. Approximately 80% of households reported drinking water from an “improved” water sources, and when accounting for in-home water treatment, nearly 93% of households consumed “safe” drinking water. Four-fifths of households used an improved sanitation facility.

In total, 1445 children aged 0–59 months were included in the AzNS. Among these children, the prevalence of stunting was below 20% nationally, and few children were wasted. Combined overweight and obesity was found in 14.0% of children, with 8.1% and 5.9% classified as overweight and obese, respectively.

Of the 1413 children aged 6–59 months recruited for the AzNS who were eligible to provide blood samples, hemoglobin concentration was measured in 1111 (78.6%). About one-quarter had some form of anemia (Table 2), while 0.5%, 7.7%, and 16.1% had severe, moderate, and mild anemia, respectively. Iron deficiency was observed in 15.0% of sampled children, and iron deficiency anemia was observed in 6.5% of children. Vitamin A and zinc deficiencies were observed in 7.8% and 11.0% of children, respectively. Of note, the vitamin A deficiency prevalence of 11.7% in urban children is statistically significant (*p* < 0.01) and higher than the 5.0% in rural children. Nearly 32% of children had some form of inflammation, with the vast majority of these children having chronic inflammation, identified by elevated AGP only.

The AzNS included 3081 non-pregnant women 15–49 years of age; of these, 2819 (91.5%) had complete anthropometric measurements, and 2706 (87.8%) had hemoglobin measurements. While very few women had undernutrition, overweight and obesity were much more common and increased with age. Anemia was found in 38.2% of women (Table 2): 1.1% of women had severe anemia, 18.1% had moderate anemia, and 19.0% had mild anemia. About one-third of women had iron deficiency, and almost one-quarter had iron deficiency anemia. About one-tenth of women had vitamin A insufficiency. A high proportion of women had inflammation. More than one-third of non-pregnant women were classified as folate deficient. Among the 1336 women tested (45.2% of all recruited women), almost 20% were vitamin B12 deficient.

Table 3 demonstrates that anemia prevalence decreased consistently as age increased in children 6 to 59 months old. In addition, boys had a higher prevalence of anemia than girls. There were no statistically significant differences among the regions nor by household wealth. Although children living in households with an unsafe water supply had a higher prevalence of anemia than those in households with a safe water supply, there was little difference in anemia prevalence by household sanitation status. There is also some suggestion, albeit not quite statistically significant, that children whose households had adequate handwashing facilities, children whose mothers were more educated, and children who had no diarrhea in the past 2 weeks had a lower prevalence of anemia. None of the indicators of complementary feeding (e.g., minimum dietary diversity, minimum meal frequency, minimum dietary adequacy, consumption of iron-rich foods) measured in this survey were statistically significantly associated with anemia. Anemia was less common in children without a recent history of lower respiratory infection. Anemia prevalence did not substantially differ by the presence of wasting, overweight, or stunting. Iron deficient children were significantly more likely to be anemic (see Table 3), and among anemic children, 28.0% were iron deficient (data not shown). On the other hand, children with vitamin A deficiency or zinc deficiency were not statistically significantly more likely to be anemic. Children with any degree of inflammation did have a higher prevalence of anemia.

As consumption of iron-rich foods in the past 24 h was measured in children 6–23 months of age, we examined if this consumption was associated with iron status. The iron deficiency prevalence among children that consumed iron-rich foods was lower (19.9%; 95% CI: 14.3, 27.1) than in those that did *not* consume iron rich foods (27.3%; 95% CI: 17.2, 40.3), but the difference was not statistically significant (*p* = 0.264, data not shown).

Anemia prevalence in women of reproductive age significantly differed by age (*p* < 0.05), residence (*p* < 0.001) and region (*p* < 0.001; Table 4). Iron, vitamin A, and folate deficiencies were significantly (*p* < 0.01) associated with anemia in women, whereas household level factors, underweight, B12 deficiency, inflammation, and lactation status were not associated with anemia.

The prevalence of inflammation (46.8%; 95% CI: 43.1, 50.6) among overweight and obese women was significantly (*p* < 0.0001) higher than the inflammation prevalence among women with normal weight (21.6%; 95% CI: 18.9, 24.5; data not shown).

Table 5 presents the separate final poisson regression models for anemia as the outcome for children and women. In children, LRI, inflammation, and iron status remained significant in the model, along with child age as a covariate. While the coefficient for age is not shown because it is a continuous variable without a meaningful relative risk, age contributed to the model with statistical significance (*p* < 0.001). Crude risk ratios and aRR showed that children with recent LRI, inflammation, and iron deficiency had 1.5- to more than 2-fold increased risk of being anemic. Iron deficiency, inflammation, and LRI accounted for about 35% of anemia in children.

In women, obesity, overweight, iron deficiency, and vitamin A insufficiency were the only variables significantly associated with anemia in the multivariate model. Whereas iron and vitamin A deficiencies were associated with an increased risk of anemia, the risk of anemia was lower in overweight and obese women. More than 40% of risk of anemia in women was explained by iron deficiency alone, while vitamin A insufficiency explained only a negligible proportion.

## 4. Discussion

### 4.1. Micronutrient Deficiencies: Prevalence

The AzNS provided critical data to estimate the prevalence of micronutrient deficiencies and the determinants of anemia. Among children, the prevalence of iron deficiency was generally low, and is classified by the WHO as “not prevalent” [15]. Child iron deficiency and iron deficiency anemia prevalences are also lower than in other countries; pooled estimates from an analysis of 23 countries with nationally representative data found that 17.3% and 9.6% of children had iron deficiency and iron deficiency anemia, respectively [27]. This suggests that iron deficiency plays a less important role in the etiology of anemia in Azerbaijani children than in children in other countries. The situation is quite different for Azerbaijani women, in whom the prevalence rates of iron deficiency and iron deficiency anemia were substantially higher. The iron status of children and women is likely driven by the consumption of dietary iron (i.e., heme and non-heme) in commonly eaten foods, as iron supplement consumption is low and no iron fortification programs exist in Azerbaijan. The AzNS data are not sufficient to corroborate this statement in women or all children. However, the consumption of iron-rich foods was assessed in children 6–23 months of age by asking about foods consumed in the past 24 h. Children consuming iron-rich foods in the past 24 h had a lower prevalence of iron deficiency (19.9%) than children who did not eat iron-rich foods (27.3%), although the difference was not statistically significant. Nonetheless, the implementation of an iron fortification program as it is currently planned by the Azerbaijan government (i.e., addition of 55 mg of iron per kilogram of flour) [28] in combination with promotion of iron supplements could reduce the iron deficiency prevalence, especially in women. However, since the bioavailability of iron-fortified foods can be adversely affected by iron absorption inhibitors (e.g., phytic acid) [29], future iron-fortification programs should be accompanied by appropriate messaging related to avoiding anti-nutrient containing foods (e.g., black tea) during meals.

Vitamin A deficiency prevalence in Azerbaijani children was low and denotes a mild public health problem [18]. In women, vitamin A insufficiency was also relatively low, however, no international thresholds exist to classify the public health significance of vitamin A insufficiency. The low prevalence of vitamin A deficiency and insufficiency is somewhat surprising since Azerbaijan has no vitamin A fortification or biofortification programs. In children 6–59 months old, the vitamin A supplementation coverage was only 58% in 2014 [30]. Thus, the vitamin A status may be due to high consumption of pro-vitamin A-rich food in most parts of the country. Nonetheless, the higher prevalence of vitamin A deficiency in urban areas may indicate that urban children consume less pro-vitamin A or vitamin A rich foods than rural children. To address this urban/rural disparity, the coverage of bi-annual vitamin A supplementation should be increased in urban areas along with programs promoting foods rich in pro-vitamin A. This is particularly relevant to children in Baku where the prevalence of vitamin A deficiency is the highest and is indicative of a moderate public health problem.

Despite its relatively poor sensitivity and vulnerability to contamination, plasma zinc is the most widely used biomarker to measure zinc status and has been judged appropriate to determine the risk of populations to develop zinc deficiency [31]. To date, only about 20 countries have national data on zinc status in children using plasma/serum zinc [32] as most estimations have been made based on dietary intake of absorbable zinc and stunting prevalence [33]. WHO, UNICEF, the International Atomic Energy Agency, and the International Zinc Nutrition Consultative Group jointly recommend implementing interventions improving zinc status if the estimate prevalence of plasma or serum zinc deficiency is greater than 20%. In Azerbaijan, zinc deficiency as well as the surrogate measure of stunting prevalence are below this cut off, thus the implementation of zinc interventions need not have first priority. Nonetheless, the planned enrichment of wheat flour with zinc (25 mg of zinc per kilogram of flour) should be sufficient to fill the gap between zinc intake and zinc requirements.

Folate and vitamin B12 deficiencies were both prevalent in Azerbaijani women, but the proportion with concurrent folate and B12 deficiency was only 5.7%. Similar to vitamin A deficiency in children, folate deficiency in women tended to be higher in urban areas, indicating lower intake of folate rich foods in urban areas. As folate and vitamin B12 deficiencies both have serious health consequences such as megaloblastic anemia or neural tube defects [34,35], efforts should be made to further reduce or eliminate these deficiencies. According to the AzNS, only 7% of women reported consuming multivitamin supplements in the past 6 months [10]. Increasing the coverage of supplementation and wheat flour fortification and biofortification [34] could help to alleviate problems associated with folate and B12 deficiencies.

### 4.2. Under- and Overweight: Prevalence

Children in Azerbaijan have relatively low levels of stunting and wasting and would be defined by WHO criteria as having problems of low public health significance. The estimated prevalence of wasting in young children in Azerbaijan is statistically indistinguishable from that in the WHO Standard Population. Also, this survey’s findings demonstrate a decline in the prevalence of stunting and wasting compared to prior assessments [36]. Despite the low overall national prevalence, stunting was somewhat more common in some regions such as Ganja-Gazakh and Lenkeran, in which estimated prevalence was about 25%. A sizeable proportion of children were overweight or obese. However, when compared to the 2006 DHS, the combined prevalence of overweight and obesity has only slightly increased from 12.9% in 2006 to 14.1% in 2013 [36].

Undernutrition in women is not of major concern in Azerbaijan. The larger problem in this group is overweight and obesity, especially in older women. The prevalence of combined overweight and obesity is comparable to that in the 2006 DHS [36]; however, the prevalence of obesity increased from 17.9% to 24.1% in the 7-year period. Although this analysis did not identify contributory factors to overweight in adult non-pregnant women, such research in the future is crucial to identify interventions to address this widespread nutrition problem. Nutrition programs should focus on raising awareness of the major health consequences of overweight and obesity.

### 4.3. Anemia Prevalence and Risk Factors in Children

The prevalence of anemia in Azerbaijani children 6–59 months of age denotes a moderate public health problem [13]. The anemia prevalence found by the 2006 DHS was 39.1% [36], suggesting a decline of about 15 percentage points during the past decade. The reduction in anemia from the 2006 DHS to the AzNS appears to be the result of a decrease in the proportion of children with moderate anemia; the proportion of children with mild and severe anemia did not change during this time period. As the 2006 DHS did not assess the proximate risk factors of anemia, it is difficult to speculate what factors could have driven this reduction in anemia between 2006 and 2013. Although the AzNS measured hemoglobin concentration in venous blood and the 2006 DHS used capillary blood, both surveys used the same device (Hb201+, HemoCue™) and comparisons of venous and capillary blood samples on this device have found similar hemoglobin concentrations [37].

The low prevalence of iron deficiency anemia in children suggests that a small proportion of anemia may be associated with iron deficiency; less than 30% of anemic children had concurrent iron deficiency. Estimates of the population attributable fraction for iron deficiency imply that iron deficiency is a factor in only one-fifth of child anemia. This estimate is similar to the findings of systematic meta-analysis, which estimated that 27% of anemia in Azerbaijani children is attributable to iron deficiency [27]. Nonetheless, iron deficiency is a strong determinant of anemia in children and produces other negative health and nutrition outcomes. As a result, it should be addressed in future nutritional programs targeting children.

These findings clearly suggest that factors other than iron deficiency are contributing to most of the anemia observed in Azerbaijani children. While no other nutritionally-related factors (e.g., vitamin A and zinc deficiencies) proved to be risk factors for anemia in children, our analysis identified that non-nutritional factors, LRI and inflammation, contributed to anemia and accounted for approximately 15% of all anemia in children. Inflammation has been associated with anemia in children in many countries [38,39], and statistically significant bivariate associations between inflammation and anemia have been observed in the neighboring Republic of Georgia [38]. Although the prevalence of inflammation in Azerbaijani children was low compared to high infection burden countries [27], our analyses illustrate that children with inflammation are more likely to develop anemia than children without inflammation.

While our findings imply that LRI in children results in a higher risk of anemia, other studies have observed the same association between these factors but have suggested that anemia in children results in greater risk of LRI [40,41,42]. However, in addition to these independent studies, a systematic review and meta-analysis of the determinants of LRI found “inconsistent” associations between anemia and LRI [43]. Nonetheless, these studies did not identify if the association between anemia and LRI is causal or due to confounding, and thus cannot ascertain the direction of the association observed. Furthermore, most studies did not detail the biological pathway in which anemia could cause LRI. In the case-control study conducted by Hussain et al. [42], the authors observed that 79% of the anemia cases had concurrent iron deficiency, and that children with LRI had significantly lower serum iron concentrations. The authors speculated that the association between iron deficiency and LRI may be due to iron’s influence on immune function [44]. Moreover, a double-blind randomized controlled trial in children found that administering iron and zinc supplementation to children for 6 months (age 6 to 12 months) reduced the rate of LRI, and that this change was not associated with increased hemoglobin levels [45]. These findings therefore suggest that the direction of the association (i.e., anemia → LRI) found by other researchers is principally due to confounding between anemia and iron deficiency, and is not relevant in areas like Azerbaijan where the iron deficiency prevalence in children is low. While our analysis is based on cross-sectional data, and therefore cannot determine causality, the existing evidence suggests that our study identified the correct direction of the association in Azerbaijani children (i.e., LRI → anemia). We hypothesize that LRI could lead to anemia in Azerbaijan by evoking the inflammatory response which slows erythropoesis or by leading to virtual iron deficiency by sequestering iron as a part of the routine response to infection [46].

Many different factors may play a contributory role in anemia, [3] and our analyses did not allow for a complete investigation of all possible risk factors for anemia. For example, inherited hemoglobin disorders interfere with erythropoesis and hemoglobin production. Azerbaijan is located in a thalassemia endemic region [47], accordingly, thalassemias may have also contributed to the anemia observed, but these conditions were not investigated by the AzNS.

### 4.4. Anemia Prevalence and Risk Factors in Non-Pregnant Women

The prevalence of anemia in non-pregnant women is classified by the WHO as a moderate public health problem [13]. Contrary to children, the anemia prevalence of non-pregnant women in the AzNS was similar to the 35.7% prevalence found by the 2006 DHS [36]. However, the DHS calculation included only women that were non-pregnant and non-breastfeeding, and would increase if breastfeeding women were included, as the anemia prevalence of breastfeeding women was notably higher (53.4%). As with children, the AzNS measured hemoglobin concentration in women in venous blood whereas the 2006 DHS used capillary blood, but as similar anemia prevalence was found, this method may not have impacted hemoglobin concentration.

In contrast to children, iron deficiency made a substantial contribution to the anemia prevalence found in Azerbaijani women. Our findings are similar to those calculated by Petry & Olofin et al., that found that 62.1% of anemia in Azerbaijani women was attributable to iron deficiency [27]. This major risk factor, plus the smaller role played by vitamin A insufficiency, suggests that the majority of anemia found in woman can be classified as nutritional anemia. While obesity has been associated with inflammation and subsequent anemia in other countries [48], and our study found similar associations between obesity and inflammation, we found no association between inflammation and anemia in women. Furthermore, our analysis found that the risk of anemia was lower in overweight and obese women, suggesting that while these over-nutrition states may result in inflammation, this chronic inflammation does not subsequently result in anemia. We did, however, find a lower prevalence of inflammation-adjusted iron deficiency in overweight and obese women: iron deficiency prevalence was 40.3%, 32.4%, and 26.6% in normal weight, overweight, and obese women, respectively (*p* < 0.001; data not shown). This may suggest that the diet of overweight and obese women is richer in iron than that of normal women, which in turn results in a lower anemia prevalence. As our study cannot identify possible reasons for this association, other studies should investigate the potential behavioral and diet-related risk factors of iron deficiency in Azerbaijani women.

## 5. Conclusions

Our analysis identified potential risk factors of anemia, but due to the cross-sectional nature of AzNS, we cannot establish causality between any outcome of interest. Nonetheless, the results of the AzNS show that anemia remains a public health problem in both children and women in Azerbaijan. Iron deficiency is strongly associated with anemia in children and women, but as the prevalence of iron deficiency is higher in women, iron deficiency contributes to a greater proportion of anemia in women than in children. While overweight and obese women are less likely to have anemia, the high prevalence of overweight and obesity among women is a public health concern. 

## Figures and Tables

**Table 1:** Household characteristics, Azerbaijan 2013.

Characteristic	Number of Households	Percentage ^a^	(95%CI) ^b^
Residence (*N* = 3926)			
Rural	2361	48.0	(42.6, 53.4)
Urban	1565	52.0	(46.6, 57.4)
Region (*N* = 3926)			
Baku	368	10.4	(9.4,11.5)
Absheron	453	12.1	(11.0, 13.4)
Aran	581	14.6	(13.4, 15.9)
Dagliq Shirvan	370	9.2	(8.7, 9.8)
Ganja-Gazakh	403	10.0	(9.5, 10.6)
Quba-Khachmaz	450	11.1	(10.7, 11.5)
Lenkeran	445	11.8	(11.3, 12.4)
Sheki-Zaqatala	449	10.8	(10.2, 11.5)
Yukhari Karabakh	407	9.9	(9.3, 10.5)
Sex of head of household (*N* = 3926)			
Male	2961	75.1	(73.1, 77.1)
Female	965	24.9	(22.9, 26.9)
Drinking water source (*N* = 3847)			
Improved	3146	81.0	(76.3, 85.0)
Unimproved	701	19.0	(15.0, 23.7)
Drinking water safety (*N* = 3886)			
Safe	3591	92.5	(90.2, 94.2)
Unsafe	295	7.5	(5.8,9.8)
Sanitation adequacy (*N* = 3919)			
Improved	3038	80.0	(76.8, 82.8)
Unimproved	881	20.0	(17.2, 23.2)

Note: The n’s are un-weighted denominators for each subgroup; subgroups that do not sum to the total have missing data. ^a^ Percentages weighted for unequal probability of selection, except distribution of regions. All results presented as percentages unless noted otherwise. ^b^ CI = confidence interval, calculated taking into account the complex sampling design.

**Table 2:** Micronutrient and anthropometric indicators of preschool children and non-pregnant women of reproductive age, Azerbaijan 2013.

Characteristic	Preschool Children ^a^			Non-Pregnant Women		
	Number of Children Tested	%/Mean/Median ^b^	(95%CI) ^c^	Number of Women Tested	%/Mean/Median ^b^	(95%CI) ^c^
Hemoglobin (g/L), mean	1111	116.3	(115.3, 117.3)	2704	121.7	(120.8, 122.6)
Anemia, % ^d^	1111	24.8	(21.6, 28.2)	2704	38.2	(35.7; 40.8)
Iron status ^e,f^						
Ferritin, (µg/L), unweighted median	1075	27.4	(26.0, 28.9)	2649	25.0	(23.3, 26.3)
Iron deficiency, %	1075	14.9	(12.4, 17.7)	2647	34.2	(31.7; 36.7)
Iron deficiency anemia, %	1099	6.7	(5.2, 8.6)	2645	23.8	(21.8; 26.1)
Vitamin A status ^e,g^						
RBP, (µmol/L), mean	1075	1.06	(1.04, 1.09)	2647	1.48	(1.45, 1.50)
Vitamin A deficiency, %	1075	7.8	(5.7; 10.7)	--	--	--
Vitamin A insufficiency, %	--	--	--	2647	10.5	(9.0, 12.1)
Zinc status						
Plasma zinc (µg/dL), mean	1046	70.9	(69.8, 72.0)	--	--	--
Zinc deficiency, % ^h^	1046	11.0	(9.0, 13.4)	--	--	--
Folate status						
Plasma folate (ηmol/L), mean	--	--	--	2582	11.7	(11.4, 12.0)
Folate deficiency, %	--	--	--	2582	35.1	(31.5; 38.9)
Vitamin B12 status						
Plasma B12 (pmol/L), mean	--	--	--	1335	273.7	(259.6, 287.8)
Vitamin B12 deficiency, %	--	--	--	1335	19.6	(16.0; 23.9)
Inflammation						
No inflammation, %	1080	68.1	(64.4; 71.6)	2647	64.1	(61.6; 66.6)
Elevated CRP only, %	1080	1.0	(0.6; 2.0)	2647	3.1	(2.3; 4.2)
Elevated CRP and AGP, %	1080	7.0	(5.3; 9.2)	2647	10.2	(8.7; 11.9)
Elevated AGP only, %	1080	23.8	(20.7; 27.3)	2647	22.6	(20.4; 25.0)
Anthropometric categories in children						
HAZ, mean	1455	−0.70	(−0.86, −0.52)	--	--	--
Stunting (≤−2 Z-scores), %	1455	18.0	(15.2, 21.3)	--	--	--
WHZ, mean	1445	0.70	(0.61, 0.79)	--	--	--
Any wasting (≤−2 Z-scores), %	1455	3.1	(2.2, 4.4)	--	--	--
Severe wasting, % ^i,j^	1455	1.1	(0.7, 1.8)	--	--	--
Moderate wasting, % ^i,j^	1455	2.0	(1.2, 3.1)	--	--	--
Normal, % ^i,j^	1455	82.8	(80.0, 85.3)	--	--	--
Overweight, % ^i,j^	123	8.2	(6.4, 10.4)	--	--	--
Obese, % ^i,j^	84	5.9	(4.6, 7.5)	--	--	--
Anthropometric categories in non-pregnant women						
BMI, mean	--	--	--	2823	26.3	(26.0, 26.6)
Severe energy deficiency,%	--	--	--	2823	0.2	(0.1, 0.5)
Moderate energy deficiency,%	--	--	--	2823	0.8	(0.5, 1.4)
At-risk of energy deficiency,%	--	--	--	2823	3.9	(3.1, 4.9)
Normal,%	--	--	--	2823	41.9	(39.4, 44.4)
Overweight,%	--	--	--	2823	29.0	(27.0, 31.0)
Obese,%	--	--	--	2823	24.2	(22.1, 26.4)

Note: The n’s are un-weighted denominators for each subgroup; subgroups that do not sum to the total have missing data. ^a^ Anthropometry indicators collected from children aged 0–59; blood biomarkers collected from children 6–59 months of age. ^b^ Percentages weighted for unequal probability of selection. ^c^ CI = confidence interval, calculated taking into account the complex sampling design. ^d^ Anemia defined in children and women defined as Hb < 110 g/L and Hb < 120 g/L, respectively. ^e^ Ferritin and RBP concentrations, and associated deficiency prevalences, corrected for inflammation according to Thurnham [14,16]. ^f^ Iron deficiency defined as serum ferritin <12 µg/L in children and <15 µg/L in women; iron deficiency anemia defined as low serum ferritin and low hemoglobin. ^g^ Vitamin A deficiency and vitamin A insufficiency defined as RBP < 0.7 µmol/L and RBP < 1.05 µmol/L, respectively. ^h^ Plasma zinc measured in non-fasting children; cut-offs to define zinc deficiency were 65 µg/L plasma zinc (blood sample taken in the morning) or 57 µg/L plasma zinc (blood sample taken in the afternoon). ^i^ For women: BMI < 16.0 severe chronic energy deficiency; 16.0–16.9 moderate chronic energy deficiency; 17.0–18.4 at-risk for energy deficiency; 18.5–24.9 normal; 25.0–29.9 overweight; ≥30.0 obese; ^j^ For children: severe wasting: WHZ < −3; moderate wasting: WHZ −3 to <−2; normal: WHZ −2 to +2; overweight: WHZ > +2 to +3; obese: WHZ > +3.

**Table 3:** Anemia prevalence in children 6–59 months by demographic characteristics and nutritional, disease, and environmental risk factors of anemia, Azerbaijan 2013.

Characteristic	Number of Children Tested	Anemia % ^a,b^ (95% CI) ^c^	Chi-Square *p*-Value ^d^
Age group (in months)			
6–11	87	40.0 (29.2, 51.8)	<0.001
12–23	209	34.8 (27.7, 42.7)	
24–35	244	23.8 (17.7, 31.2)	
36–47	285	20.6 (15.2, 27.4)	
48–59	286	18.2 (13.3, 24.2)	
Sex			
Male	617	28.2 (23.5, 33.4)	<0.05
Female	494	20.5 (16.8, 24.8)	
Residence			
Urban	371	21.4 (17.0, 26.7)	0.080
Rural	738	27.3 (23.2, 31.7)	
Region			
Baku	50	14.1 (6.7, 27.4)	0.347
Absheron	90	22.9 (13.3, 36.5)	
Aran	182	31.5 (24.7, 39.2)	
Daghligh Shirvan	119	29.8 (21.1, 40.3)	
Ganja-Gazakh	113	20.9 (15.2, 27.9)	
Guba-Khachmaz	122	25.6 (16.3, 37.6)	
Lankaran	149	26.1 (18.1, 35.9)	
Sheki-Zaqatala	129	29.4 (20.8, 39.8)	
Yukhari Garabakh	157	20.8 (14.5, 29.1)	
Wealth Quintile			
Lowest	186	31.2 (23.4, 40.1)	0.424
Second	222	23.9 (17.8, 31.4)	
Middle	235	23.5 (17.6, 30.7)	
Fourth	254	25.7 (19.5, 33.1)	
Highest	211	20.9 (14.6, 28.8)	
Household water source			
Unsafe	219	29.5 (23.4, 36.5)	0.050
Safe	865	22.7 (19.2, 26.6)	
Household sanitation			
Inadequate	300	26.4 (20.6, 33.3)	0.524
Adequate	805	24.1 (20.5, 28.1)	
Household handwashing place has soap and water			
Yes	66	23.7 (20.5, 27.4)	0.053
No	1014	36.8 (24.0, 51.8)	
Mother’s Education			
Basic secondary or less	257	31.5 (25.0, 38.7)	0.079
Some or completed secondary	377	26.4 (21.2, 32.4)	
Higher	174	19.6 (13.2, 28.3)	
Diarrhea in the past 2 weeks			
Yes	67	34.3 (22.7, 48.1)	0.097
No	1044	24.0 (20.8, 27.6)	
Child had lower respiratory infection			
Yes	63	39.8 (25.6, 55.8)	<0.05
No	1036	23.8 (20.6, 27.3)	
Minimum dietary diversity ^e^			
Yes	158	36.2 (29.0, 45.2)	0.647
No	132	33.1 (23.7, 44.0)	
Minimum meal frequency ^e^			
Yes	160	37.8 (29.1, 47.3)	0.789
No	114	35.9 (26.4, 46.7)	
Minimum dietary adequacy ^e^			
Yes	51	47.2 (32.0, 63.0)	0.133
No	234	33.7 (26.5, 41.9)	
Consumption of iron-rich foods ^e^			
Yes	177	31.9% (24.5, 40.3)	0.171
No	107	41.6 (30.5, 53.7)	
Wasting/Overweight			
Wasted (WHZ ≤ −2)	27	26.8 (11.9, 49.8)	0.529
Normal (WHZ > −2 to ≤+2)	900	25.3 (21.6, 29.3)	
Overweight/obese (WHZ > +2)	142	20.5 (14.1, 28.6)	
Stunting			
Yes	171	23.3 (16.9, 31.3)	0.730
No	909	24.7 (21.1, 28.6)	
Iron status			
Deficient (sF < 12 µg/l)	185	46.0 (37.1, 55.1)	<0.001
Sufficient (sF ≥ 12 µg/l)	889	20.5 (17.1, 24.3)	
Vitamin A status			
Deficient (RBP < 0.70 μmol/L)	60	27.0 (15.2, 43.3)	0.692
Sufficient (RBP ≥ 0.70 μmol/L)	1014	24.0 (20.7, 27.7)	
Zinc status			
Deficient	122	31.6 (22.6, 42.2)	0.133
Sufficient	927	23.8 (20.5, 27.5)	
Inflammation			
None	761	20.9 (17.5, 24.8)	<0.01
CRP and/or AGP elevated	313	31.4 (25.5, 38.1)	

Note: The n’s are the un-weighted numbers of children in each subgroup; subgroups that do not sum to the total have missing data. ^a^ Percentages weighted for non-response and survey design. ^b^ Anemia defined as hemoglobin <110 g/L adjusted for altitude. ^c^ CI = confidence interval, adjusted for cluster sampling design. ^d^ Chi-square *p*-value < 0.05 indicates that the variation in the values of the subgroup are significantly different from all other subgroups. ^e^ Dietary consumption indicators only collected in children 6–23 months of age.

**Table 4:** Anemia prevalence in non-pregnant women 15–49 years by demographic characteristics and nutritional, disease, and environmental risk factors of anemia, Azerbaijan 2013.

Characteristic	Number of Women Tested	Anemia % ^a,b^ (95% CI) ^c^	Chi-Square *p*-Value ^d^
Age Group (in years)			
15–19	371	36.5 (30.0, 43.4)	<0.05
20–24	449	46.2 (40.2, 52.2)	
25–29	426	41.7 (36.4, 47.3)	
30–34	357	36.8 (31.2, 42.8)	
35–39	328	32.3 (26.1, 39.1)	
40–44	361	35.4 (29.8, 41.4)	
45–49	412	35.3 (30.1, 41.8)	
Residence			
Urban	972	43.0 (39.0, 47.0)	0.001
Rural	1731	34.3 (31.3, 37.5)	
Region			
Baku	184	45.5 (39.3, 51.8)	<0.001
Absheron	256	36.8 (30.4, 43.8)	
Aran	457	42.0 (36.5, 47.8)	
Daghligh Shirvan	268	35.9 (28.9, 43.6)	
Ganja-Gazakh	297	28.0 (23.6, 32.8)	
Guba-Khachmaz	301	39.4 (33.3, 45.9)	
Lankaran	340	29.9 (24.8, 35.4)	
Shaki-Zaqatala	318	37.8 (32.5, 43.4)	
Yukhari Garabakh	283	35.5 (28.5, 43.1)	
Woman Education			
Basic secondary or less	786	38.7 (34.1, 43.4)	0.754
Some or completed secondary	1442	37.4 (33.8, 41.1)	
Higher	475	39.8 (34.1, 45.8)	
Own agricultural land			
Yes	1477	34.1 (30.8, 37.6)	0.001
No	1211	42.0 (38.6, 45.5)	
Wealth Quintile			
Lowest	477	40.0 (34.4, 45.7)	0.401
Second	542	41.7 (35.5, 48.2)	
Middle	560	34.3 (29.7, 39.3)	
Fourth	561	38.8 (33.4, 44.4)	
Highest	553	37.5 (33.0, 42.2)	
Household water source status			
Unsafe	500	33.5 (27.5, 40.1)	0.119
Safe	2146	39.3 (36.6, 42.1)	
Household sanitation status			
Inadequate	644	40.5 (34.9, 46.4)	0.352
Adequate	2053	37.6 (34.9, 40.3)	
Household handwashing place has soap and water			
Yes	2439	37.8 (35.1, 40.5)	0.753
No	190	39.3 (30.6, 48.7)	
Underweight			
Underweight (BMI < 18.5)	127	36.7 (25.8, 49.1)	0.135
Normal (BMI 18.5–24.9)	1135	46.1 (42.3, 49.8)	
Overweight/obese			
Obese (BMI ≥ 30.0)	658	29.6 (25.1, 34.6)	<0.001
Overweight (BMI 25.0–29.9)	778	34.6 (30.5, 38.8)	
Normal (BMI 18.5–24.9)	1135	46.1 (42.3, 49.8)	
Iron status			
Deficient (sF < 12 µg/L)	929	69.8 (65.4, 73.8)	<0.001
Sufficient (sF ≥ 12 µg/L)	1716	21.9 (19.3, 24.8)	
Vitamin A insufficient			
Insufficient (RBP < 1.05 μmol/L)	274	59.5 (51.3, 67.3)	<0.001
Sufficient (RBP ≥ 1.05 μmol/L)	2371	35.8 (33.1, 38.5)	
Folate status			
Deficient (pF < 10 nmol/L)	912	44.7 (40.1, 49.4)	<0.001
Sufficient (pF ≥ 10 nmol/L)	1668	35.2 (32.3, 38.2)	
B12 status			
Deficient (pB12 < 150 pmol/L)	265	36.6 (30.1, 43.6)	0.455
Sufficient (pB12 ≥ 150 pmol/L)	1070	39.6 (35.8, 43.7)	
Inflammation			
None	1725	38.3 (34.9, 41.8)	0.978
CRP and/or AGP elevated	920	38.1 (34.4, 42.2)	
Currently lactating			
Yes	206	43.5 (34.5, 52.9)	0.346
No	1640	39.0 (36.0, 42.2)	

Note: The n’s are un-weighted denominators for each subgroup; subgroups that do not sum to the total have missing data. ^a^ Percentages weighted for non-response and survey design. ^b^ Anemia defined as hemoglobin <110 g/L adjusted for altitude. ^c^ CI = confidence interval, adjusted for cluster sampling design. ^d^ Chi-square *p*-value < 0.05 indicates that the variation in the values of the subgroup are significantly different from all other subgroups.

**Table 5:** Crude and adjusted relative risk of anemia and population attributable fraction in children 6–59 months old and non-pregnant women 15–49 years of age, Azerbaijan 2013.

Characteristic	Category	*N*	Crude (Bivariate Analysis)		Adjusted (Poisson Regression)		Population Attributable Fraction ^b^
			Relative Risk	95% CI	Relative Risk	95% CI	
**Children 6–59 months ^a^ (*N* = 1062)**							
Child had LRI	Yes	63	1.6	(1.1, 2.2)	1.6	(1.1, 2.1)	3.4%
	No	1036	referent	-	referent	-	
Inflammation	Yes	313	1.3	(1.1, 1.6)	1.5	(1.2, 1.9)	13.6%
	No	761	referent	-	referent	-	
Iron status	Deficient	185	2.7	(2.2, 3.3)	2.6	(2.1, 3.1)	17.6%
	Not deficient	889	referent	-	referent	-	
**Non-pregnant women 15–49 years (*N* = 2516)**							
Obesity	Obese	658	0.64	(0.55, 0.73)	0.74	(0.65, 0.84)	−7.1%
	Normal	1128	referent	-	referent	-	
Overweight	Overweight	780	0.75	(0.67, 0.85)	0.84	(0.75, 0.93)	−5.5%
	Normal	1128	referent	-	referent	-	
Iron status	Deficient	929	3.3	(3.0, 3.7)	3.2	(2.8, 3.6)	43.2%
	Not deficient	1718	referent	-	Referent	-	
Vitamin A status	Insufficient	274	1.6	(1.5, 1.8)	1.3	(1.2, 1.4)	3.7%
	Not insufficient	2373	referent	-	referent	-	

^a^ Child’s age in months as a continuous covariate was included in the child regression model. ^b^ Calculated using relative risk from poisson regression.
